# Capillary stalling after traumatic brain injury: an emerging microcirculatory contributor to secondary brain injury

**DOI:** 10.3389/fneur.2026.1971528

**Published:** 2026-10-06

**Authors:** Xiaolong Lei, Yan Yang, Xiaokun Yang, Kai Tao

**Affiliations:** 1 Department of Clinical Medicine, North Sichuan Medical College, Nanchong, Sichuan, China; 2 Department of Emergency Medicine, The General Hospital of Western Theater Command, Chengdu, Sichuan, China

**Keywords:** capillary stalling, cerebral hypoxia, cerebral microcirculation, neurocritical care, neurovascular unit, pericytes, secondary brain injury, traumatic brain injury

## Abstract

Secondary brain injury after traumatic brain injury (TBI) has traditionally been understood and managed through the lens of intracranial pressure, cerebral perfusion pressure, and global cerebral blood flow. Yet a substantial proportion of patients continue to exhibit persistent tissue hypoxia and metabolic crisis even when these macrocirculatory parameters appear adequately controlled. Capillary stalling—the transient or sustained interruption of red blood cell transit within individual or focal capillary networks—may represent an under-recognized microcirculatory disorder that conventional macrovascular monitoring cannot capture. This review synthesizes evidence supporting capillary stalling as a potentially distinct and reversible component of post-traumatic microvascular failure. We examine candidate mechanisms of capillary flow interruption after TBI, including endothelial injury and glycocalyx disruption, leukocyte plugging, pericyte contraction, platelet activation and microthrombosis, red blood cell dysfunction, and edema-associated extravascular compression. These mechanisms can generate marked capillary flow heterogeneity, impair oxygen extraction, and contribute to metabolic crisis, neuroinflammation, and secondary neuronal injury. We further discuss why conventional bedside monitoring—including intracranial pressure, cerebral perfusion pressure, global cerebral blood flow, brain tissue oxygenation, and microdialysis—may fail to detect capillary-level flow abnormalities, and we review established and emerging detection strategies from experimental two-photon microscopy to clinical surrogates. Finally, we evaluate potential microcirculation-targeted therapeutic avenues. Direct evidence of capillary stalling in human TBI remains limited; accordingly, it should currently be viewed as a biologically plausible and testable contributor to secondary brain injury rather than an established dominant driver.

## 1 Introduction

### 1.1 Traumatic brain injury and the unresolved burden of secondary injury

Traumatic brain injury (TBI) remains a leading cause of death and long-term disability worldwide (1, 2), affecting millions of individuals each year across all age groups and income settings. The pathology of TBI is classically partitioned into a primary injury—the immediate mechanical disruption of neurons, glia, and vessels occurring at the moment of impact—and a secondary injury, a delayed cascade of ischemia, hypoxia, excitotoxicity, inflammation, and edema that evolves over hours to days (3, 4). It is this secondary phase that has proven to be both the dominant determinant of neurological outcome (5) and the principal window for therapeutic intervention. Among the many components of secondary injury, cerebral ischemia and hypoxia stand out as potent and treatable drivers of poor outcome (6), and their relationship to patient prognosis has been recognized for decades. Yet despite improvements in trauma systems, monitoring, and neurocritical care, the translation of this knowledge into reliably better outcomes has remained frustratingly incomplete.

### 1.2 Limitations of the conventional macrocirculatory paradigm

Modern management of severe TBI rests on a small set of macrocirculatory targets: intracranial pressure (ICP), cerebral perfusion pressure (CPP), mean arterial pressure (MAP), and, when measured, global or hemispheric cerebral blood flow (CBF). Guidelines-based protocols that titrate treatment to keep ICP below threshold and CPP within target ranges (7, 8) have become the backbone of neurocritical care. The assumption underlying this paradigm is that maintaining adequate driving pressure (9) across the cerebral vascular bed will ensure sufficient perfusion and oxygenation of the parenchyma. However, a growing body of clinical observation contradicts this assumption. Adequate macrocirculatory parameters do not necessarily guarantee adequate microvascular perfusion or tissue oxygenation. Many patients in whom ICP and CPP are successfully controlled nonetheless exhibit regional brain tissue hypoxia (low brain tissue oxygen tension, PbtO₂), elevated lactate–pyruvate ratios, and neurochemical signs of metabolic crisis on microdialysis (10, 11). This paradox raises the first core question of this review: why do some TBI patients develop ongoing cerebral hypoxia and metabolic disturbance despite apparently satisfactory control of ICP and CPP?

### 1.3 Capillary stalling as an overlooked component of secondary brain injury

One plausible answer lies not at the level of the large conducting vessels but at the level of the capillary bed—the site where the functional exchange of oxygen and substrate with tissue actually occurs. Capillary stalling refers to the transient or sustained cessation of red blood cell (RBC) transit within an individual capillary or a small cluster of capillaries (12, 13), while the surrounding network continues to perfuse. It is important to distinguish capillary stalling from global hypoperfusion, arterial occlusion, vasospasm, and classical structural microthrombosis (14). Capillary stalling is characteristically focal, spatially heterogeneous, dynamic, potentially reversible, and often recurrent, and it can coexist with entirely normal flow in adjacent capillaries and with normal global CBF. Because each stalled capillary removes a discrete microdomain from the oxygen-diffusion field (15, 16), even a modest fraction of stalled capillaries can produce substantial tissue hypoxia out of proportion to any measurable change in whole-brain perfusion. In the setting of TBI, the combination of mechanical vascular injury, endothelial and pericyte dysfunction, inflammation, and coagulopathy provides an unusually fertile substrate for this phenomenon. This review examines capillary stalling as a potentially under-recognized microcirculatory mechanism linking traumatic vascular injury (17, 18) to persistent cerebral hypoxia, metabolic crisis, and secondary brain injury. We first outline the organization and regulation of the cerebral microcirculation, then review the concept and current evidence for capillary stalling after TBI, dissect the molecular and cellular mechanisms that drive it, and trace the pathway from stalled capillaries to tissue injury. We conclude by addressing why conventional monitoring may miss this disorder, which detection strategies are available or emerging, and whether restoring capillary flow represents a realistic therapeutic ambition in neurocritical care.

### 1.4 Literature search and selection

This narrative review was informed by a structured search of PubMed/MEDLINE and Web of Science, performed up to the date of manuscript preparation, using combinations of terms including ‘traumatic brain injury’, ‘capillary stalling’, ‘capillary transit time heterogeneity’, ‘cerebral microcirculation’, ‘pericyte’, ‘no-reflow’, ‘leukocyte plugging’, and ‘microvascular dysfunction’. Reference lists of retrieved articles were screened for additional sources, and priority was given to original research, systematic reviews, and authoritative clinical guidelines. Because this is a narrative rather than a systematic review, formal inclusion and exclusion criteria and quantitative synthesis were not applied, and the selection of included sources reflects the authors’ judgement of relevance and methodological quality.

## 2 Cerebral microcirculation in the injured brain

### 2.1 Organization of the cerebral microcirculation

The cerebral circulation is organized as a hierarchical network in which pial arteries give rise to penetrating arterioles that descend into the parenchyma and branch into an extraordinarily dense capillary plexus before draining into venules and veins. The true functional exchange surface is the capillary bed, whose endothelial cells are surrounded by basement membrane, occasional contractile pericytes, and astrocytic endfeet (19). This structural ensemble—endothelial cell, pericyte, astrocyte endfoot, and the intervening basal lamina and glycocalyx—is collectively termed the neurovascular unit (20–22), reflecting the intimate functional coupling between neuronal activity, glial signaling, and vascular behavior. Two features of the capillary bed deserve emphasis. First, its surface area is enormous, and the diffusion distance from any neuron to its nearest capillary is short, making the capillary the rate-limiting site for oxygen and glucose delivery. Second, the capillary is not a passive conduit: endothelial cells sense and transduce hemodynamic and inflammatory signals, pericytes can regulate capillary caliber (23, 24), and the endothelial glycocalyx—a luminal layer of glycosaminoglycans and glycoproteins—modulates vascular permeability, mechanotransduction, and leukocyte–endothelial interactions (25). In addition to mediating exchange, the cerebral vasculature provides a conduit for interstitial fluid clearance. Fluid within the basement membranes of capillaries and arteries can move in directions distinct from—and in some circumstances opposite to—luminal blood flow, a pathway termed intramural periarterial drainage that is increasingly recognized as relevant to perivascular clearance and neurovascular health (26, 27). This directional dissociation between abluminal fluid movement and intraluminal blood flow is an important part of the microenvironment in which capillary stalling occurs and may itself be perturbed after injury.

The hierarchical organization of the cerebral vasculature and the dimensional relationships among key vascular and cellular components relevant to capillary stalling are illustrated in Figure 1.

**Figure 1:**
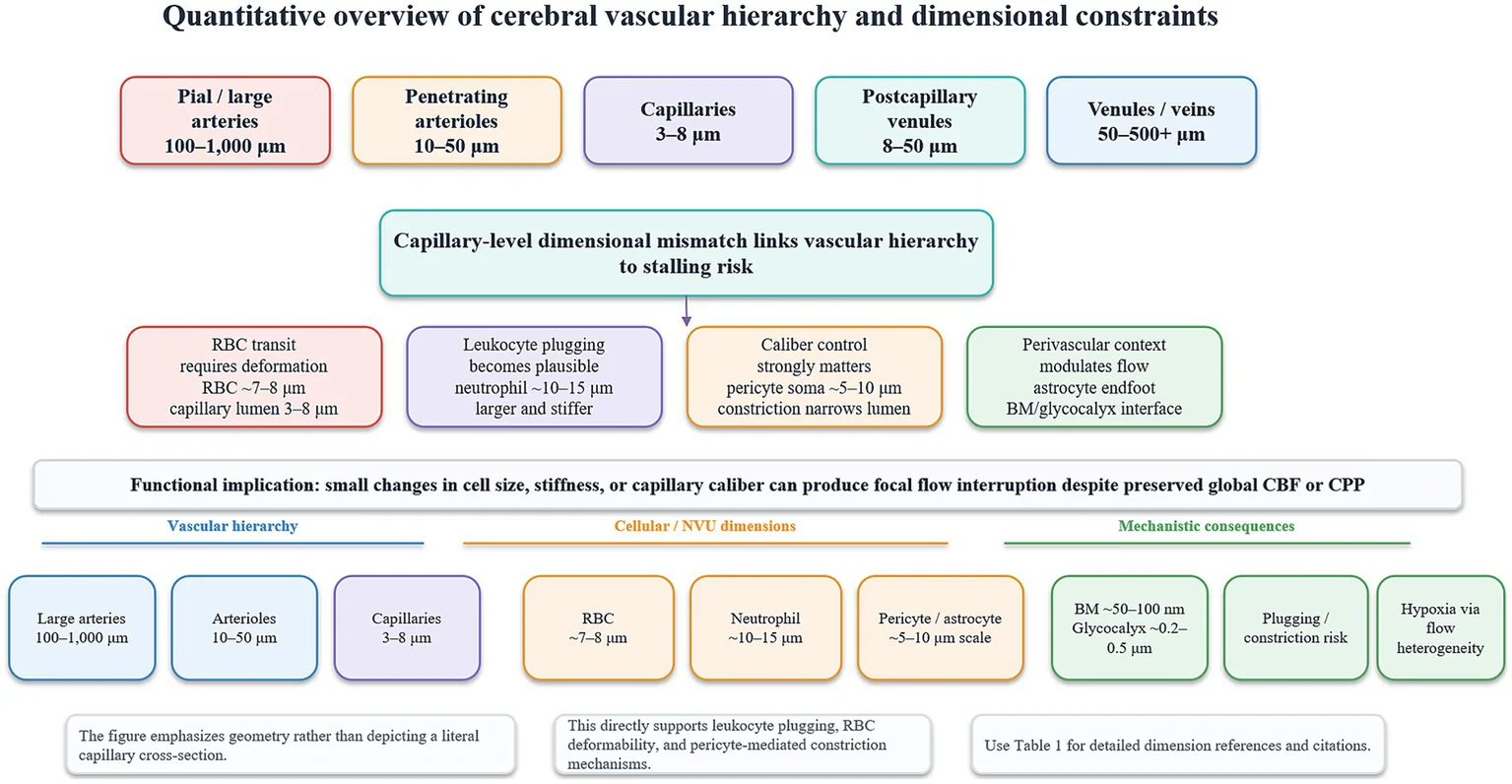
Quantitative overview of the cerebral vascular hierarchy and dimensional constraints relevant to capillary stalling. The cerebral vascular tree spans large pial arteries, penetrating arterioles, capillaries, postcapillary venules, and veins, with progressively smaller vessel calibers toward the capillary bed. Capillaries have luminal diameters of approximately 3–8 μm, comparable to the diameter of red blood cells (RBCs; ~7–8 μm), which therefore require substantial deformation during transit. Neutrophils are larger (~10–15 μm) and less deformable, increasing their potential to obstruct narrow capillary segments. Pericytes, astrocyte endfeet, the basement membrane, and the endothelial glycocalyx further shape the local neurovascular microenvironment and capillary caliber. These dimensional relationships help explain why modest changes in cell size, stiffness, vascular tone, or extravascular pressure can produce focal capillary flow interruption, increased flow heterogeneity, and impaired oxygen extraction even when global cerebral blood flow or cerebral perfusion pressure is preserved.

Approximate diameters of the principal vascular and cellular constituents of the cerebral circulation, and their relevance to capillary stalling, are summarized in Table 1.

**Table 1:** Approximate dimensions of vascular and cellular components relevant to capillary stalling.

Component	Approximate dimension	Relevance to capillary stalling
Pial and large cerebral arteries	100–1,000 μm	Conducting vessels upstream of the capillary bed
Penetrating arterioles	10–50 μm	Feed the capillary bed; principal site of vasospasm
Capillaries	3–8 μm	Site of stalling; lumen diameter comparable to a red blood cell
Postcapillary venules and veins	8–500 μm	Drainage; principal site of leukocyte adhesion and emigration
Red blood cells	~7–8 μm (≈2.5 μm thick)	Must deform to transit the narrowest capillaries
Leukocytes (neutrophils)	~10–15 μm	Largest circulating cells; can plug narrow capillary segments
Pericytes	Soma ~5–10 μm; processes ~50–100 μm	Regulate capillary caliber and red blood cell transit
Astrocyte endfeet	~5–10 μm thick	Ensheath capillaries; component of the neurovascular unit
Endothelial basement membrane and glycocalyx	Basement membrane ~50–100 nm; glycocalyx ~0.2–0.5 μm	Luminal–abluminal interface and perivascular clearance pathway

### 2.2 Regulation of capillary blood flow and oxygen delivery

Oxygen delivery to brain tissue is governed not only by the total flow delivered but by its distribution in time and space. Within a capillary network, the relevant parameters are red blood cell velocity, capillary diameter, and the residence time of blood within each capillary (28)—collectively determining capillary transit time. Because oxygen extraction is a saturable, diffusion-limited process, tissue oxygenation depends critically on the uniformity of transit times across the capillary network. When transit times are homogeneous and matched to metabolic demand, oxygen extraction is efficient. When transit-time heterogeneity increases—with some capillaries carrying blood too rapidly to allow extraction and others too slowly or not at all—the efficiency of oxygen extraction falls even if total CBF is unchanged. This principle, formalized in the capillary transit-time heterogeneity (CTH) framework, explains why regional oxygen delivery can deteriorate without any measurable fall in global blood flow (15, 29, 30). It also provides the theoretical basis for the central claim of this review: that a relatively small number of stalled capillaries, by creating shunts through well-perfused adjacent capillaries and flow gaps through stalled ones, can meaningfully impair tissue oxygenation (16, 31).

### 2.3 Microvascular disruption after TBI

Traumatic injury produces an immediate and dramatic perturbation of the cerebral microvasculature. Mechanical deformation of the brain applies direct shear and tensile forces to vessel walls, causing endothelial injury and disruption of the blood–brain barrier (BBB) (32–34). This mechanical disruption is increasingly conceptualized within the framework of traumatic axonal and/or microvascular injury (TAMVI), a term that unifies axonal pathology with direct small-vessel trauma—including microhemorrhage and vascular congestion—produced by inertial forces (35). Because TAMVI entails direct injury to the microvasculature itself, with endothelial disruption, pericyte detachment, and capillary wall damage, it is likely to be intimately linked to capillary stalling, providing an anatomical substrate on which dynamic capillary flow failure can arise. The injured endothelium loses its antithrombotic and anti-adhesive phenotype, the glycocalyx is shed, and leukocyte adhesion molecules are exposed on the luminal surface (36, 37). Concurrently, perivascular pericytes are exposed to an abnormal biochemical environment—elevated reactive oxygen species, disturbed calcium handling, and vasoactive peptides such as endothelin-1 (38, 39)—that can shift them toward a contracted state. The systemic coagulation system is activated at sites of tissue factor exposure, generating fibrin-rich microthrombi in downstream capillaries (40, 41), while activated platelets and neutrophils physically obstruct the narrowest vessels. The net functional consequence is microvascular flow heterogeneity: some capillaries remain normally perfused, others carry sluggish flow, and a minority become completely stalled. Capillary stalling may therefore represent one of the functional manifestations of this post-traumatic microvascular failure (16), and understanding its determinants requires closer examination of the concept and its evidence base.

## 3 Capillary stalling after traumatic brain injury: concept and evidence

### 3.1 Definition and phenotypes of capillary stalling

Capillary stalling can be defined operationally as the interruption of red blood cell transit through a single capillary or a focal cluster of capillaries, lasting from seconds to minutes or longer, in the absence of overt occlusion of the feeding arteriole. Direct *in vivo* observations, particularly using two-photon microscopy, have revealed that stalling is not a binary phenomenon (12, 42) but spans a spectrum of phenotypes. Transient stalling describes brief cessations of RBC flux that resume spontaneously (43); intermittent stalling is characterized by repeated stop–start cycles; and persistent stalling denotes prolonged flow cessation exceeding minutes. Partial obstruction presents as the passage of a single-file RBC column at markedly reduced velocity or as plasma skimming in which plasma continues to flow while RBCs are trapped. Complete cessation of RBC transit represents the most severe end of the spectrum. These phenotypes are not mutually exclusive and may coexist within the same network (18), reflecting different underlying mechanisms—leukocyte plugging, pericyte-mediated constriction, microthrombotic obstruction, or rheological failure—which we dissect in Section 4.

### 3.2 Experimental evidence

The most direct evidence that capillary stalling occurs in the injured brain comes from animal models studied with high-resolution imaging. Two-photon and intravital microscopy through cranial windows have allowed investigators to track RBC motion through individual cortical capillaries (28, 44) with millisecond temporal resolution and capillary-level spatial resolution. In experimental ischemia and in models of cerebral injury, these studies document the cessation of RBC flux in individual capillaries, pericyte-associated narrowing of the capillary lumen, leukocyte plugging at capillary branch points, and patchy, regional capillary perfusion failure that spares neighboring vessels (24, 45, 46). In the context of reperfusion after transient ischemia, a substantial fraction of capillaries remain non-perfused despite successful recanalization of the upstream artery—a phenomenon termed the no-reflow phenomenon—and imaging has directly attributed this to a combination of pericyte constriction and luminal obstruction (14, 47, 48). Capillary stalling has likewise been synthesized as a common microvascular pathway to stroke susceptibility and poor recovery, extending its relevance beyond TBI to the broader field of cerebrovascular disease (49). Although analogous direct imaging studies in TBI are less numerous, experimental TBI demonstrates regional microcirculatory perfusion deficits and functional microvascular abnormalities (16, 32, 50) that are compatible with capillary-level flow interruption but do not, by themselves, establish the frequency or dynamics of capillary stalling after trauma.

### 3.3 Evidence from human TBI

Direct visualization of capillary stalling in human TBI remains limited (16). In contrast to the experimental setting, the clinical environment does not permit routine optical imaging of single capillaries in the injured human cortex. Current evidence in patients is therefore largely indirect. Studies using bedside techniques and tissue assays have documented regional hypoperfusion in injured brain regions, reductions in brain tissue oxygen tension (PbtO₂), microdialysis-detected metabolic crisis, and histopathological evidence of microvascular abnormalities and post-traumatic microthrombi (10, 11, 40, 51, 52) in tissue obtained at surgery or autopsy. These observations are entirely consistent with—and most plausibly explained by—the occurrence of capillary-level flow failure, but they do not prove it directly. We emphasize this evidentiary boundary deliberately: the concept that capillary stalling occurs in human TBI is biologically plausible and mechanistically supported, but its definitive confirmation awaits imaging approaches capable of resolving single capillaries in the clinical setting. Acknowledging this limitation strengthens rather than weakens the argument, by defining precisely what is established and what remains to be demonstrated.

### 3.4 Capillary stalling versus related microvascular phenomena

Because capillary stalling overlaps with several well-known microvascular concepts, it is essential to delineate the boundaries. Microthrombosis refers to structural, occlusive intravascular plugs composed of platelets, fibrin, and entrapped blood cells (40); stalling, by contrast, can occur in the absence of any structural obstruction and is fundamentally a dynamic flow phenomenon. Vasospasm describes constriction of pial and penetrating arterioles (50)—vessels an order of magnitude larger than capillaries—whereas stalling involves the capillary segment itself and may be driven by pericyte constriction rather than smooth muscle spasm. No-reflow is the failure of perfusion to be restored after a period of ischemia despite arterial recanalization (14, 47); it is an end-state description of a particular temporal context, whereas stalling is a description of ongoing, potentially reversible flow dynamics that can also occur in the primary injury phase. Finally, global hypoperfusion denotes a reduction in total CBF (51), whereas stalling is inherently focal and heterogeneous, and can occur against a background of normal global flow. The practical consequence is that stalling is not reducible to any of these entities; it occupies its own conceptual niche (15), with its own mechanisms, its own detection requirements, and potentially its own therapeutic vulnerabilities.

The principal conceptual distinctions among capillary stalling and related microvascular phenomena are summarized in Table 2.

**Table 2:** Capillary stalling and related microvascular phenomena: conceptual distinctions.

Phenomenon	Definition	Anatomical scale	Predominant nature	Reversibility	Prototypical context	Key references
Capillary stalling	Transient or sustained cessation of RBC transit in individual capillaries	Single capillary or focal cluster	Dynamic flow failure	Potentially reversible and recurrent	Post-ischemia; TBI; inflammation	(12, 13, 16, 18, 42, 43)
Microthrombosis	Structural occlusive plug of platelets, fibrin, and blood cells	Capillaries to venules	Structural occlusion	Requires lysis or clearance	Post-TBI; ischemia–reperfusion	(32, 40, 65)
Vasospasm	Constriction of pial and penetrating arterioles	Arterioles (10–300 μm)	Dynamic smooth-muscle constriction	Potentially reversible	Post-hemorrhage; subarachnoid hemorrhage	(47, 50)
No-reflow	Failure of perfusion to be restored after ischemia despite arterial recanalization	Capillary bed	End-state of ischemia–reperfusion	Variable; may be irreversible	Ischemia–reperfusion	(14, 46–48)
Global hypoperfusion	Reduction in total or regional CBF	Whole brain/hemisphere/region	Hemodynamic failure	Reversible with pressure support	Raised ICP; hypotension	(51, 83)

CBF, cerebral blood flow; RBC, red blood cell.

## 4 Mechanisms driving capillary stalling after TBI

### 4.1 Endothelial injury and glycocalyx disruption

The endothelial surface is the first barrier that mechanical trauma perturbs, and its dysfunction is central to the initiation of capillary flow failure. Shear and tensile forces during impact directly injure endothelial cells, and the damaged endothelium assumes a pro-adhesive, pro-thrombotic, and pro-inflammatory phenotype (32). A particularly consequential early event is the shedding of the endothelial glycocalyx, the luminal glycosaminoglycan–proteoglycan layer that normally repels leukocytes and platelets and transduces shear stress into vasodilatory nitric oxide (NO) signaling. Circulating fragments of the glycocalyx core protein syndecan-1 are elevated in trauma patients and correlate with endothelial injury, inflammation, coagulopathy, and mortality (37, 53). Once the glycocalyx is lost, adhesion molecules that are normally concealed—intercellular adhesion molecule-1 (ICAM-1), vascular cell adhesion molecule-1 (VCAM-1), and the selectins—become exposed on the luminal surface (25, 36), enabling leukocyte tethering, rolling, and firm adhesion, and von Willebrand factor (VWF) released from injured endothelium supports platelet adhesion at the capillary level (54, 55). The immediate functional consequence is a disturbance of capillary flow: leukocytes that would normally be swept through the network now arrest and obstruct the narrowest vessels, converting the biochemical injury of the endothelium into the mechanical event of capillary obstruction. This sequence—mechanical stress, endothelial activation, glycocalyx shedding, adhesion-molecule exposure, leukocyte adhesion, and capillary flow disturbance—constitutes one of the most direct mechanistic routes from trauma to stalling (33).

### 4.2 Neutrophil plugging and leukocyte–endothelial adhesion

Leukocytes are physically the largest cells that must deform to negotiate the capillary bed, and their behavior is a strong candidate driver of post-traumatic stalling. Traumatic injury releases a flood of damage-associated molecular patterns (DAMPs) from injured neurons, glia, and the extracellular matrix, which activate the endothelium and recruit neutrophils (56, 57) to the injured brain. In the microvasculature, this recruitment translates directly into mechanical events: neutrophils rolling on selectins, firmly adhering via integrin–ICAM-1 interactions, and in many cases arresting within—and thereby plugging—capillary segments (12, 45). Direct imaging in non-traumatic experimental models has demonstrated leukocyte adhesion and capillary plugging, with upstream flow stalling that can reverse when leukocytes disengage (12, 45, 46). Experimental TBI studies, in turn, establish robust neutrophil recruitment and a pathogenic contribution of neutrophils to edema and tissue injury (56–58), although they do not by themselves prove capillary stalling. This observation supports a conceptually important point: post-traumatic inflammation may impair cerebral perfusion not only biochemically, through cytokines and vasoactive mediators, but also mechanically, through the physical obstruction of the smallest vessels by leukocytes (56). The relative stiffness of neutrophils—particularly activated neutrophils with reorganized actin cytoskeletons—further impairs their ability to deform through capillaries (46), compounding the effect of adhesion. Targeting this mechanism may therefore offer a direct route to restoring capillary patency.

### 4.3 Pericyte contraction and capillary constriction

Pericytes are the mural cells of the capillary bed, positioned along the capillary wall where they are uniquely placed to regulate capillary caliber (23, 59) and, consequently, RBC transit. In the healthy brain, pericytes receive relaxing signals—including NO—and maintain capillary patency. After injury, the pericyte micro-environment deteriorates in several convergent ways: reactive oxygen species (ROS) accumulate, intracellular calcium homeostasis is disturbed, endothelin-1 is released in excess, RhoA/ROCK signaling is activated, and NO bioavailability falls (48, 60, 61). The resulting imbalance shifts pericytes toward a contracted state, narrowing the capillary lumen at discrete points along its length. Because the capillary lumen is barely wider than the RBC, even a pericyte-mediated reduction in diameter of a few percent can transform smooth RBC passage into complete transit failure (24, 31). This mechanism is supported by direct experimental evidence in ischemia, where pericyte constriction has been shown to be a major cause of capillary no-reflow despite upstream recanalization, and where inhibition of pericyte contraction restores capillary flow (14, 48). Translating these observations to TBI, the pericyte emerges as both a plausible mediator of post-traumatic stalling and a highly attractive therapeutic target (39, 62): unlike the irreversible obstruction of a formed thrombus, pericyte-mediated constriction is potentially reversible if the biochemical environment can be corrected (13). It must be acknowledged, however, that this model is not universally accepted; some studies argue that capillary caliber is governed principally by upstream arteriolar smooth muscle contractility rather than pericyte tone (63), a controversy that should be borne in mind when interpreting pericyte-centered therapeutic claims.

### 4.4 Platelet activation, microthrombosis, and immunothrombosis

Traumatic brain injury is characterized by an intense and early activation of the coagulation system. Tissue factor released from the injured brain and exposed at disrupted vascular sites initiates thrombin generation and fibrin formation; activated platelets adhere to exposed collagen and to endothelium-bound VWF (40); and the fibrin-rich plugs that form in the microcirculation constitute the well-recognized post-traumatic microthrombi. Histopathological studies have long documented microthrombi in the cerebral microvasculature after fatal TBI, and their burden correlates with regions of ischemia and infarction (32, 40). In recent years, this coagulation–inflammation axis has been reconceptualized through the lens of immunothrombosis, in which neutrophils and neutrophil extracellular traps (NETs) (64, 65)—latticeworks of DNA, histones, and antimicrobial proteins released by dying neutrophils—scaffold platelet adhesion and fibrin formation and entrap RBCs (65, 66). Neutrophil–platelet interactions within the microvasculature can therefore convert a purely inflammatory event into a thrombotic one and vice versa (41, 67). Importantly, capillary stalling may frequently represent a pre-thrombotic, dynamic state: stalled flow promotes local coagulation activation, and a stalled capillary is fertile ground for the formation of a lasting microthrombus. Conversely, immunothrombotic occlusion may represent the structural end-point of stalling that fails to reverse. We therefore propose that immunothrombotic capillary stalling may represent a bridging concept between dynamic flow interruption and structural microvascular occlusion after TBI (64), although we emphasize that this formulation remains a hypothesis to be tested rather than an established conclusion.

### 4.5 Red blood cell dysfunction and impaired deformability

A largely overlooked contributor to post-traumatic stalling is the red blood cell itself. The circulation of RBCs through capillaries requires continuous and rapid deformation, and any impairment of deformability converts RBCs from the most deformable elements of blood into potential occluders. After trauma, systemic oxidative stress injures the RBC membrane, reducing deformability and increasing aggregation (68); altered plasma viscosity and the inflammatory milieu further impair the rheological properties of blood (36). Hemolysis releases cell-free hemoglobin, which avidly scavenges NO and thereby removes a key relaxing stimulus from pericytes and endothelium (69, 70)—an effect that can both constrict capillaries and promote platelet and leukocyte adhesion. The result is a self-reinforcing rheological failure: RBCs that are less deformable and more aggregated are more likely to be trapped in pericyte-narrowed or leukocyte-occupied capillaries, and each trapped cell further slows the flow that might dislodge it. This red-cell-centered perspective adds an important layer of novelty to the stalling concept because it identifies a component of blood itself—rather than the vessel wall alone—as a determinant of capillary patency, with corresponding therapeutic implications for the rheological and antioxidant environment.

### 4.6 Edema and extravascular capillary compression

Finally, the damaged microvasculature is embedded within a tissue that is itself swelling. Blood–brain barrier disruption produces vasogenic edema, and the accumulation of fluid in the interstitial space raises local tissue pressure (34, 71–73). In regions of edema, the increased extravascular pressure compresses capillaries from outside, narrowing their lumina and converting normal transit into sluggish flow and, ultimately, stalling. This extravascular mechanism is distinct from the luminal mechanisms described above, yet it compounds them: a capillary already narrowed by pericyte contraction or partially obstructed by a leukocyte is far more vulnerable to external compression (72). The relationship is reciprocal and forms what may be termed a stalling–edema vicious cycle—edema compresses capillaries, stalled capillaries produce hypoxia, and hypoxia-driven BBB failure and cell swelling generate further edema (74). This positive-feedback structure suggests that microvascular compression is not a static consequence of edema but an active participant in the propagation of secondary injury, and that interventions aimed at the edematous tissue compartment may secondarily relieve capillary compression and restore flow.

The major mechanistic drivers of capillary stalling after TBI and their proposed therapeutic targets are summarized in Table 3.

**Table 3:** Mechanisms contributing to capillary stalling after traumatic brain injury.

Mechanism	Primary effectors	Key mediators	Effect on capillary flow	Representative therapeutic target	Key references
Endothelial injury and glycocalyx disruption	Endothelial cells; luminal glycocalyx	Syndecan-1 shedding; ICAM-1/VCAM-1; selectins; VWF	Loss of anti-adhesive surface; adhesion of leukocytes and platelets	Glycocalyx preservation (hydrocortisone, antithrombin)	(25, 32, 36, 37, 53, 55)
Leukocyte (neutrophil) plugging	Neutrophils; activated endothelium	DAMPs; integrins (CD11/CD18); selectins; ICAM-1	Mechanical capillary obstruction; upstream stalling	Anti-adhesion (anti-CD11d); selectin blockade	(12, 45, 46, 56–58)
Pericyte contraction	Pericytes; capillary mural cells	ROS; ET-1; Ca2+; RhoA/ROCK; reduced NO	Capillary lumen narrowing; RBC transit failure	ROCK inhibition (fasudil); ET receptor antagonism	(23, 24, 39, 48, 60, 61, 63)
Platelet activation and microthrombosis	Platelets; coagulation cascade; neutrophils	Tissue factor; thrombin; fibrin; VWF; NETs	Structural microvascular occlusion	NET degradation (DNase); antiplatelet agents	(40, 41, 54, 64–67)
Red blood cell dysfunction	RBCs	Oxidative injury; reduced deformability; cell-free hemoglobin; NO scavenging	Impaired capillary transit; aggregation and trapping	Rheological and antioxidant strategies	(36, 68–70)
Edema-associated extravascular compression	Astrocytes; BBB; interstitial space	Vasogenic edema; raised tissue pressure	Extravascular capillary compression; sluggish flow	Osmotherapy; BBB stabilization	(34, 71–74)

DAMP, damage-associated molecular pattern; ET-1, endothelin-1; NET, neutrophil extracellular trap; NO, nitric oxide; RBC, red blood cell; ROCK, Rho-associated protein kinase; VWF, von Willebrand factor.

## 5 From capillary stalling to secondary brain injury

### 5.1 Capillary flow heterogeneity and microregional hypoperfusion

If capillary stalling is to be regarded as pathologically significant, it must produce tissue injury through mechanisms that are not captured by measures of total flow. The key insight is that the problem after TBI is often not a reduction in global CBF but a maldistribution of flow (16, 47). Within an affected microdomain, some capillaries perfuse normally, others carry sluggish flow, and a subset is completely stalled. This produces marked spatial heterogeneity in perfusion at a scale far below that resolved by standard perfusion imaging (15). Neurons served by stalled capillaries are effectively disconnected from the oxygen and glucose supply, while their neighbors are served normally—yet neither condition is visible to global or regional measurements that average across thousands of capillaries. Capillary flow heterogeneity is thus the functional signature of stalling, and it is this heterogeneity, rather than hypoperfusion per se, that converts a modest loss of capillary patency into disproportionate tissue injury.

### 5.2 Impaired oxygen extraction and tissue hypoxia

The oxygen-transfer consequences of flow heterogeneity are most clearly understood through the capillary transit-time heterogeneity framework. When transit times are uniform and matched to metabolic demand, oxygen extraction is maximal (15, 75). Stalling introduces two distinct inefficiencies. First, slow or stalled capillaries lengthen transit time locally and eliminate extraction in the microdomains they serve. Second—and less intuitively—fast transit through the remaining patent capillaries shunts blood through without allowing time for oxygen extraction, a diffusion-limitation effect that reduces extraction even in well-perfused regions (15, 31). The combined result is that overall oxygen extraction efficiency falls and regional tissue hypoxia develops despite a maintained or even elevated global CBF. This mechanism offers one possible explanation for a persistent clinical paradox: brain tissue can remain hypoxic even when macrocirculatory parameters appear adequate (16), because oxygen extraction may be limited by microcirculatory disorganization. It also provides a rationale for why increasing driving pressure or global flow alone may fail to correct tissue hypoxia when flow distribution is abnormal.

### 5.3 Metabolic crisis and mitochondrial dysfunction

When oxygen delivery falls below the demands of oxidative phosphorylation, the metabolic consequences unfold in a characteristic sequence. Reduced O₂ delivery suppresses mitochondrial oxidative phosphorylation, depleting ATP and forcing the tissue toward anaerobic glycolysis. Lactate production rises, pyruvate is consumed preferentially, and the lactate-to-pyruvate ratio (LPR)—a widely used bedside index of the redox state of the tissue—increases (11, 52). A persistently elevated LPR defines the metabolic crisis that is a hallmark of severe TBI and is strongly associated with poor outcome (11, 52). It is important to recognize that metabolic crisis is not a surrogate for stalling; rather, it is a tissue-level readout of impaired energy metabolism that can arise from several mechanisms. If capillary stalling produces microregional hypoxia after TBI, it could contribute to the metabolic derangements detected by microdialysis, providing a testable link between capillary-level flow failure and bedside metabolic monitoring (76).

### 5.4 Neuroinflammation, excitotoxicity, and cellular injury

Hypoxia and metabolic crisis do not occur in isolation; they ignite the cellular injury cascades that constitute secondary brain injury. Hypoxic neurons release ROS and DAMPs, which activate resident microglia and recruit additional inflammatory cells, perpetuating the neuroinflammatory response (56, 77). Energy failure impairs the ATP-dependent membrane pumps that maintain ion gradients; the resulting membrane depolarization releases glutamate into the synaptic cleft in excess, and the sustained activation of glutamate receptors floods neurons with calcium (78). Calcium overload activates proteases, lipases, and nucleases, producing neuronal and glial injury and, ultimately, cell death (6). These cascades are well-established components of secondary brain injury. Their proposed linkage to stalling is indirect: capillary-level flow interruption could sustain microregional hypoxia and thereby amplify metabolic stress, excitotoxicity, and inflammation. Under this model, stalling would function as a potential amplifier of cellular injury rather than a proven independent determinant of neurological outcome.

### 5.5 A proposed capillary stalling–hypoxia–inflammation feedback loop

A central hypothesis of this review is that capillary flow interruption, hypoxia, and inflammation may form a self-amplifying loop. Capillary stalling could promote microregional hypoxia; hypoxia can generate oxidative stress and DAMP release; these signals can intensify inflammation and endothelial activation; and leukocyte adhesion or other microvascular changes may further impair capillary flow (12, 13, 18). In this proposed feedback model, an initially focal and potentially reversible microcirculatory disturbance could expand the territory of tissue at risk. The model identifies several candidate intervention points—restoring capillary flow, limiting leukocyte adhesion, or reducing injurious inflammation—but each requires direct testing in TBI. It also predicts that persistent microvascular dysfunction may promote further flow abnormalities, a hypothesis that can be examined with longitudinal capillary-level imaging. Direct testing of this feedback architecture will be important to determine whether disrupting the cycle can meaningfully limit secondary injury after TBI. The proposed mechanistic framework linking capillary stalling to microregional hypoxia, neuroinflammation, and progressive microvascular dysfunction after TBI is summarized in Figure 2.

**Figure 2:**
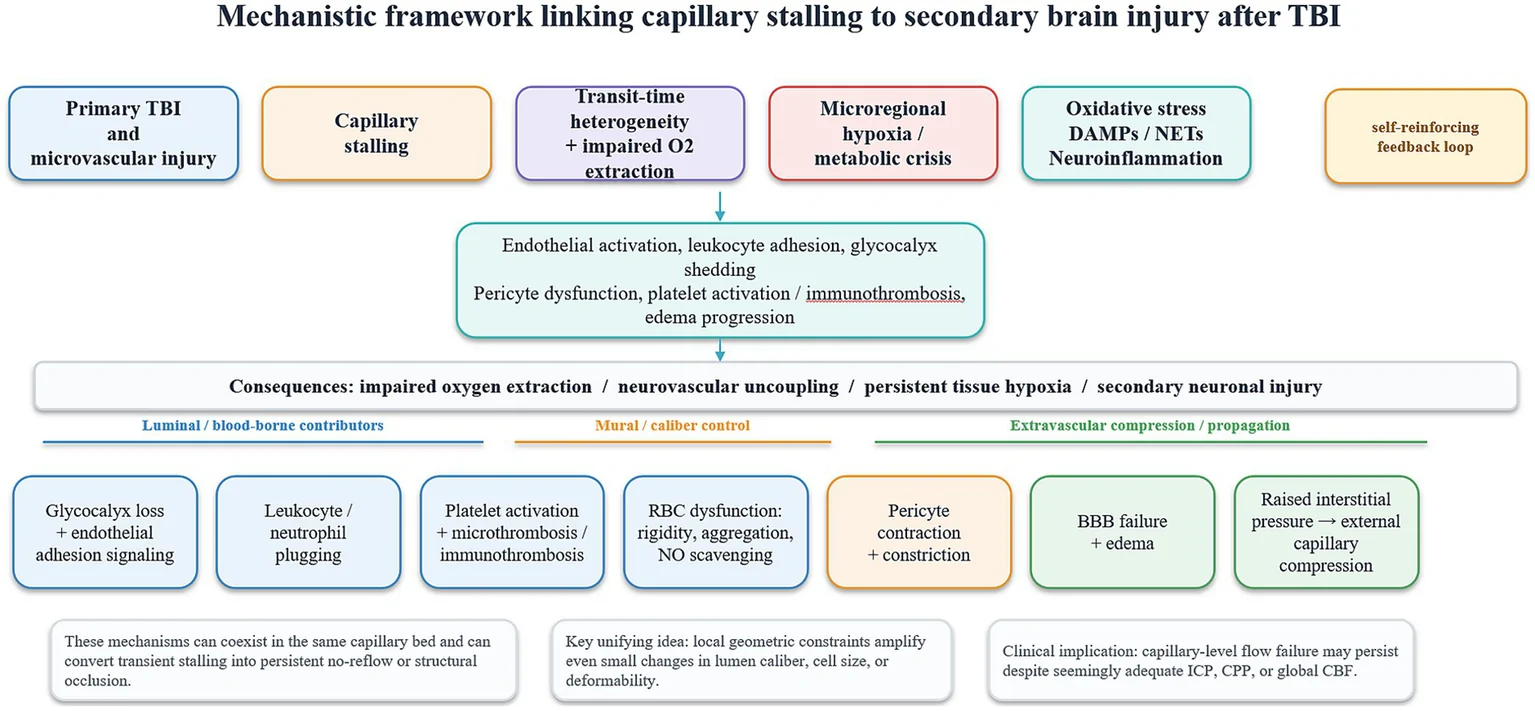
Mechanistic framework linking capillary stalling to secondary brain injury after traumatic brain injury. Traumatic brain injury (TBI) can initiate microvascular dysfunction that promotes capillary stalling through multiple interacting mechanisms, including endothelial and glycocalyx injury, leukocyte and neutrophil plugging, platelet activation and microthrombosis, red blood cell (RBC) dysfunction, pericyte-mediated constriction, blood–brain barrier (BBB) failure, edema, and increased interstitial pressure. Capillary stalling increases capillary transit-time heterogeneity and impairs oxygen extraction, contributing to microregional hypoxia, metabolic crisis, oxidative stress, damage-associated molecular pattern (DAMP) release, neutrophil extracellular trap (NET) formation, and neuroinflammation. These processes further promote endothelial activation, leukocyte adhesion, immunothrombosis, pericyte dysfunction, and edema progression, creating a self-reinforcing feedback loop that may sustain secondary microvascular and neuronal injury despite apparently adequate global cerebral perfusion.

## 6 The clinical blind spot: why conventional TBI monitoring may miss capillary stalling

### 6.1 Intracranial pressure

Intracranial pressure is the cornerstone of TBI monitoring, and its importance is beyond dispute (7). However, ICP is fundamentally a measurement of the pressure environment of the cranium—the product of intracranial volume and compliance—and it carries no direct information about the state of the microcirculation. A normal or controlled ICP does not indicate that individual capillaries are patent, that RBCs are transiting, or that any particular microregion is perfused (79, 80). Compensatory mechanisms, the redistribution of cerebrospinal fluid, and the topographic heterogeneity of injury mean that significant microcirculatory failure can coexist with acceptable ICP (81). ICP monitoring is therefore necessary but insufficient for assessing microvascular perfusion: it detects the pressure consequences of swelling and mass lesions but does not directly determine whether individual capillary networks remain functionally perfused.

### 6.2 Cerebral perfusion pressure

Cerebral perfusion pressure, defined as the difference between mean arterial pressure and intracranial pressure, is a pressure-derived estimate of the driving force for cerebral blood flow (9). It is a core target of guideline-based management in severe TBI, and individualized CPP optimization may be associated with better physiological control and outcomes (9, 82). Yet CPP is not a direct measurement of functional microvascular perfusion (10). It describes the pressure available to drive flow but cannot determine how resistance is distributed across arterioles, capillaries, and venules. If capillary resistance is increased by constriction, cellular plugging, edema, or microthrombosis, an acceptable CPP could coexist with impaired microvascular flow. CPP should therefore be interpreted as a macrocirculatory target rather than evidence that capillary perfusion is intact.

### 6.3 Global cerebral blood flow

Measurements of cerebral blood flow, whether by invasive probes, transcranial Doppler-derived surrogates, or perfusion imaging, generally average signals over vascular territories containing many microvessels (51). Such averaging can obscure spatial heterogeneity at the capillary scale. For example, a hypothetical region in which some capillaries are non-perfused while others carry compensatory higher flow could retain a near-normal mean CBF despite marked local flow heterogeneity. Regional perfusion abnormalities reported after TBI are consistent with this possibility (83, 84), but conventional CBF measurements do not establish capillary stalling directly. The key clinical question is therefore whether macro–microcirculatory dissociation occurs often enough, and with sufficient prognostic relevance, to justify additional microvascular assessment.

### 6.4 Brain tissue oxygenation

Brain tissue oxygen tension (PbtO₂), measured with an invasive intraparenchymal probe, is the most direct clinical index of whether the local microcirculation is actually delivering oxygen (85). Low PbtO₂ identifies tissue at risk and is independently associated with poor outcome after severe TBI, and PbtO₂-guided therapy is increasingly incorporated into multimodal management (10, 86, 87). Yet PbtO₂ also has inherent limitations as a detector of stalling. The probe samples a small, fixed volume of tissue—a few tens of cubic millimeters—and is exquisitely local; it cannot detect stalling in regions remote from the catheter. Moreover, PbtO₂ integrates oxygen supply and demand and reflects the net outcome of microvascular delivery, cellular consumption, and diffusion distance; it therefore detects the tissue-level consequence of stalling rather than stalling itself, and cannot distinguish a diffusion limitation produced by flow heterogeneity from a supply failure produced by hypoperfusion. PbtO₂ is thus a valuable sentinel for local oxygen-delivery failure, but it cannot determine whether capillary stalling, regional hypoperfusion, diffusion limitation, mitochondrial dysfunction, or another mechanism is responsible.

### 6.5 Cerebral microdialysis

Cerebral microdialysis samples extracellular fluid from a small brain region and measures metabolites of energy metabolism—including glucose, lactate, pyruvate, the lactate-to-pyruvate ratio, and glutamate (76, 88)—providing a continuous readout of local metabolic state. Microcirculatory flow failure could contribute to patterns of low glucose, elevated LPR, and rising glutamate, but these abnormalities are not specific to capillary stalling and may also arise from mitochondrial dysfunction, regional hypoperfusion, seizures, or other processes (52, 89, 90). Microdialysis therefore offers a sensitive metabolic readout of tissue distress rather than a direct measure of microvascular patency. When interpreted together with CPP, PbtO₂, and perfusion imaging, it may help identify patients in whom tissue metabolism remains abnormal despite apparently acceptable macrocirculatory targets.

## 7 Detecting capillary stalling: from experimental imaging to clinical surrogates

### 7.1 Two-photon microscopy

The reference experimental method for detecting and characterizing capillary stalling is two-photon microscopy, which allows direct, repeated visualization of individual cortical capillaries *in vivo* (28, 44), with measurement of RBC velocity in single vessels, identification of leukocyte plugging, and visualization of pericyte behavior. Its principal strength is direct observation rather than inference: temporal dynamics (transient versus persistent) can be followed over minutes to hours through cranial windows, and cellular mechanisms can be resolved. Its limitations are equally clear: it requires optical access, is restricted to superficial cortical regions accessible to the microscope, is invasive and immobile, and remains primarily an experimental technique. For chronic, longitudinal preparations specifically, long-term cranial-window implantation can induce tissue scarring and a local inflammatory response—including gliosis—that may themselves alter the microcirculation being imaged, a consideration that should temper interpretation of prolonged post-injury studies. Two-photon imaging therefore serves as a reference method for defining the phenomenon and validating candidate surrogates, while routine clinical use in TBI is not currently feasible. Its near-term role is to establish mechanistic ground truth and to evaluate therapies before clinical translation (12, 42).

### 7.2 Laser speckle and optical imaging

Laser speckle contrast imaging (LSCI) derives maps of relative blood flow from the blurring of laser speckle patterns caused by moving RBCs (91), and can image flow over a field of view of square millimeters to centimeters with high temporal resolution and no dye. It is widely used experimentally to characterize cortical perfusion dynamics (92), and its rapid acquisition allows changes in microregional blood flow to be followed over time. The principal limitations are its penetration depth—LSCI sees only the most superficial cortical layers—and its lack of single-capillary resolution in many implementations, so it reports microregional rather than truly single-capillary flow. Nevertheless, LSCI’s ability to visualize the patchy, heterogeneous perfusion failure that characterizes stalling makes it a valuable experimental bridge between single-capillary microscopy and clinically feasible approaches.

### 7.3 Optical coherence tomography angiography

Optical coherence tomography angiography (OCTA) uses the temporal decorrelation of OCT signals to image flowing blood, achieving micrometre-scale resolution of vascular networks without exogenous contrast (93). Unlike two-photon microscopy, OCTA does not require fluorophores and can image through the thinned skull or cranial window, and advances in system speed and signal processing have brought single-capillary-level imaging of cerebral flow within reach (94). The capacity to resolve individual capillaries and to detect segments with absent or intermittent flow suggests that OCTA-based approaches could eventually provide non-dye, high-resolution mapping of capillary perfusion and stalling in experimental and, potentially, clinical settings (42, 95)—for example through craniotomy windows or thinned bone. While considerable technical development and validation remain, OCTA represents one of the most promising directions for translating stalling detection from the bench toward the bedside.

### 7.4 CT and MR perfusion approaches

Conventional CT and MR perfusion techniques—CT perfusion, dynamic susceptibility contrast (DSC) MRI, dynamic contrast-enhanced (DCE) MRI, and arterial spin labeling (ASL)—are the workhorses of clinical cerebral perfusion assessment (84, 96) and can detect regional abnormalities in blood flow, blood volume, and transit time after TBI. Their power is in whole-brain coverage and clinical availability; their limitation is resolution. These methods average flow over voxels that contain many capillaries and cannot resolve the single-capillary scale at which stalling occurs. They can, however, reveal regional microcirculatory abnormalities—patchy areas of low perfusion, elevated mean transit time, or reduced blood volume—that are consistent with and may partially reflect microvascular flow failure (83, 97, 98). In this sense, advanced perfusion imaging can raise the suspicion of stalling at the regional level, and, when combined with physiological surrogates, can help identify patients in whom the macrocirculation appears normal but the microcirculation is failing (51, 99).

### 7.5 Bedside physiological surrogates

In the intensive care unit, stalling must currently be inferred from bedside surrogates. PbtO₂ and cerebral microdialysis detect the tissue-level consequences of oxygen-delivery failure, as discussed in Section 6 (10, 76). Near-infrared spectroscopy (NIRS) offers non-invasive cerebral oximetry and, in principle, continuous monitoring of regional tissue oxygenation (100, 101), though its depth and spatial resolution are limited and its relationship to single-capillary events is indirect. Transcranial Doppler (TCD) measures flow velocity in large intracranial arteries and, together with autoregulation monitoring (e.g., assessment of pressure reactivity or of autoregulatory indices) (102, 103), provides information about the reactivity of the macrocirculation rather than the patency of individual capillaries (82, 104). The rationale for combining these tools is that discordance between apparently adequate macrocirculatory parameters and persistent tissue hypoxia or metabolic crisis may be compatible with microcirculatory dysfunction. Such a multimodal pattern can identify patients for further study, but it should not be regarded as a validated signature of capillary stalling until it is compared with direct or high-resolution measures of capillary flow (105, 106).

### 7.6 Circulating microvascular biomarkers

An entirely different avenue toward detecting microvascular failure is the blood itself. Circulating markers of endothelial injury and glycocalyx degradation—syndecan-1, angiopoietin-2, von Willebrand factor, ICAM-1, and VCAM-1—and markers of NET formation are elevated after TBI and correlate with endothelial activation, inflammation, and coagulopathy (37, 55, 65, 107, 108). These biomarkers cannot localize stalling and are not specific to the capillary bed, and their relationship to directly imaged stalling has not been established. They should therefore be regarded as emerging and exploratory tools rather than validated detectors of stalling (53). Nevertheless, their appeal is considerable: they are readily accessible, repeatable, and could, in principle, provide a systemic readout of the intensity of endothelial–microvascular injury and of the response to microcirculation-targeted therapy. Combined with imaging and physiological surrogates, circulating biomarkers may eventually contribute to a composite index of microvascular failure in the injured brain.

The principal experimental and clinical approaches for detecting microvascular failure and capillary stalling after TBI are summarized in Table 4.

**Table 4:** Approaches for detecting microvascular failure and capillary stalling in TBI.

Modality	Parameter measured	Direct detection of stalling?	Coverage/resolution	Bedside availability	Limitations	Key references
Two-photon microscopy	Single-capillary RBC velocity; leukocyte plugging; pericyte tone	Yes (reference standard)	Single capillary; cortical window	Experimental only	Invasive; cranial window; shallow depth	(12, 28, 42, 44)
Laser speckle contrast imaging	Microregional flow maps	Partial	~100 μm; superficial cortex	Experimental	Limited depth; not single-capillary	(91, 92)
OCT angiography	Capillary-level flow decorrelation	Emerging	Near-capillary; contrast-free	Experimental; potential clinical use	Requires optical access; skull thinning	(42, 93–95)
CT and MR perfusion (CTP, DSC, DCE, ASL)	Regional CBF, CBV, MTT; perfusion heterogeneity	No (regional surrogate)	mm–cm voxels; whole brain	Clinical (portable for CT)	Averages over many capillaries	(83, 84, 96–99)
Brain tissue oxygen tension (PbtO₂)	Local tissue oxygen tension	Indirect (tissue consequence)	~mm3 focal	Clinical (invasive)	Local sampling; integrates supply and demand	(10, 85, 86)
Cerebral microdialysis	Glucose, lactate, pyruvate, LPR, glutamate	Indirect (metabolic consequence)	~mm3 focal	Clinical (invasive)	Detects metabolic result, not stalling itself	(11, 52, 76, 88)
NIRS, TCD, autoregulation monitoring	Regional oximetry; large-artery velocity; pressure reactivity	Indirect	Regional / large vessels	Clinical (non-invasive)	Macrocirculatory; not capillary-level	(100–104)
Circulating microvascular biomarkers	Syndecan-1, VWF, Ang-2, ICAM-1, VCAM-1, NET markers	Indirect (endothelial injury)	Systemic	Clinical (blood)	Exploratory; not specific to stalling	(37, 53, 55, 65, 107, 108)

ASL, arterial spin labeling; CBF, cerebral blood flow; CBV, cerebral blood volume; CTP, CT perfusion; DCE, dynamic contrast-enhanced; DSC, dynamic susceptibility contrast; LPR, lactate-to-pyruvate ratio; LSCI, laser speckle contrast imaging; MTT, mean transit time; NIRS, near-infrared spectroscopy; OCTA, optical coherence tomography angiography; PbtO₂, brain tissue oxygen tension; TCD, transcranial Doppler.

## 8 Therapeutic implications: can capillary flow be restored?

### 8.1 Targeting leukocyte adhesion and neutrophil plugging

If neutrophil adhesion and plugging contribute to capillary flow failure, modulating leukocyte–endothelial interactions is a plausible therapeutic strategy. Preclinical studies have reported benefit from selectin-directed approaches in hypoxic–ischemic or ischemic models (109), and CD11d/CD18 integrin blockade reduced tissue injury and improved neurological outcomes in experimental TBI (110, 111). Translation, however, requires caution: the Enlimomab Acute Stroke Trial of anti-ICAM-1 therapy did not improve outcome and was associated with more adverse events (112, 113). These findings emphasize that adhesion pathways also participate in host defense and immune regulation, so broad systemic blockade can introduce substantial risks. A more realistic TBI strategy may therefore require transient, cell-selective, or locally targeted modulation that improves microvascular patency without producing clinically important immunosuppression.

### 8.2 Targeting pericyte-mediated capillary constriction

Pericyte-mediated constriction is a particularly attractive mechanistic target because it is dynamic and potentially reversible. Pharmacological strategies that shift the pericyte away from contraction include inhibition of the endothelin pathway, augmentation of NO signaling, modulation of intracellular calcium, and inhibition of the RhoA/ROCK pathway—for example with ROCK inhibitors such as fasudil, which has shown benefit in experimental cerebral ischemia (60, 61, 114). If pericyte constriction contributes to stalling after TBI, these agents could restore capillary caliber and RBC transit without the hemorrhagic risks associated with antithrombotic therapy (13, 48). The key requirement is to establish, first, that pericyte constriction occurs in the relevant capillary population after TBI and, second, that reversing it improves tissue oxygenation and outcome—both of which are tractable experimental questions.

### 8.3 Modulating NETs and microthrombosis

The immunothrombotic arm of stalling raises the possibility of therapies that dismantle NETs or inhibit microthrombus formation, such as DNase to degrade NET scaffolds, or agents that interfere with platelet activation or fibrin formation (65, 67). The central dilemma here is clinical and immediate: in patients with traumatic intracranial hemorrhage, promoting reperfusion with antithrombotic or NET-directed therapy could increase the risk of hemorrhage expansion. Any strategy that targets microthrombosis must therefore be weighed against its bleeding risk (54, 64), and the favorable risk profile would require microvascular-selective or temporally restricted intervention—administered after bleeding has stabilized, or directed specifically at the microcirculatory compartment—rather than systemic anticoagulation. This tension between reperfusion and hemorrhagic risk is a defining challenge for the therapeutic program outlined in this review.

### 8.4 Protecting the endothelial glycocalyx

Preserving the endothelial glycocalyx offers a preventive rather than restorative strategy. Because glycocalyx shedding exposes the adhesion molecules and pro-thrombotic surfaces that initiate stalling, interventions that stabilize the endothelium and protect the glycocalyx—such as anti-inflammatory agents, sulfated glycosaminoglycans, or agents that reduce glycocalyx-degrading enzyme activity—could blunt the entire cascade at its source (115, 116). The clinical appeal of glycocalyx protection is that it acts upstream of the divergent inflammatory, thrombotic, and rheological mechanisms of stalling, and it is a strategy already under investigation in the broader fields of trauma and sepsis (36, 117). The experimental challenge is to demonstrate that glycocalyx preservation translates into maintained capillary patency and preserved tissue oxygenation after TBI.

### 8.5 Reducing edema-associated microvascular compression

Because extravascular edema compresses capillaries, therapies that reduce cerebral edema or stabilize the blood–brain barrier may secondarily relieve capillary compression and restore flow. Established interventions such as osmotherapy—mannitol and hypertonic saline—reduce brain water and intracranial pressure, and barrier-stabilizing agents may limit the fluid extravasation that drives vasogenic edema (72, 73, 118). The distinctive perspective offered by the stalling framework is that these therapies should be evaluated not only for their effect on ICP but also for their capacity to restore capillary patency: a treatment that lowers pressure without restoring microvascular flow would be expected to be incomplete. This reframing ties the edema compartment to the microcirculatory compartment and suggests that edema management and microcirculatory preservation are complementary, not competing, goals.

### 8.6 Toward microcirculation-guided neurocritical care

The therapeutic implication of this framework is not to replace established ICP- and CPP-guided care, but to test whether an additional microcirculatory layer can identify patients with persistent tissue-level oxygen-delivery failure despite apparently adequate macrocirculatory targets (87, 106). In future studies, this could involve integrating PbtO₂, cerebral microdialysis, regional perfusion imaging, and validated markers of endothelial or microvascular injury rather than relying on any single surrogate. Such multimodal patterns would first need prospective validation against direct or high-resolution measures of microvascular dysfunction before they could guide treatment. The decisive test is whether identifying and reversing capillary-level perfusion failure improves neurological outcome; until that is demonstrated in rigorous clinical studies, microcirculation-guided care should remain a research framework rather than a standard-management recommendation.

## 9 Discussion

### 9.1 Is capillary stalling a driver, marker, or consequence of secondary brain injury?

The central interpretive question is whether capillary stalling is a cause, a consequence, or merely a marker of secondary brain injury. Three positions can be defended. First, stalling could be a driver: by producing microregional hypoxia, it could directly initiate the metabolic crisis and cellular injury cascades described in Section 5. Second, stalling could be a consequence: it might arise only in tissue already damaged by the primary injury or by more upstream mechanisms of secondary injury, and might therefore be an epiphenomenon with little independent pathogenic weight. Third, and most plausibly, stalling could be an amplifier. The available evidence suggests it is neither the sole cause nor a pure result, but rather a mechanism that, once initiated, intensifies hypoxia and inflammation and thereby amplifies injury produced by other processes. This amplifier view is consistent with the feedback-loop architecture proposed in Section 5.5, and it carries a distinctive practical implication: even if stalling is not the initiating event, interrupting it could still attenuate the amplification of injury—making it a legitimate therapeutic target even in the absence of proof that it is the primary driver.

### 9.2 How strong is the current evidence in TBI?

It is important to grade the evidence honestly. There is strong evidence that the cerebral microcirculation is severely impaired after TBI: microvascular injury, endothelial dysfunction, pericyte dysfunction, glycocalyx degradation, and microthrombosis are all well documented in experimental models and, to a significant degree, in human tissue and clinical studies (23, 32, 37, 40). There is emerging evidence for capillary stalling as a distinct dynamic flow phenomenon (12, 13), derived largely from direct imaging in non-traumatic experimental models and supported in TBI by evidence of microvascular flow heterogeneity, microthrombosis, and impaired oxygen delivery. There is, by contrast, limited direct evidence in human TBI: as emphasized in Section 3.3, direct visualization of capillary stalling in patients has not yet been achieved (16), and the human data consist of indirect and correlative observations. This grading matters because it defines the boundary between what the review can claim and what remains hypothesis. The claim that stalling is a major, clinically hidden driver of secondary injury in patients must be presented as a well-motivated hypothesis awaiting direct confirmation, rather than as an established fact.

### 9.3 Translational gap between experimental and human TBI

The leap from experimental models to patients is particularly wide in this field, and the reasons are worth making explicit. In animal models, injury is typically controlled and standardized, optical access is achieved through a cranial window, and the microcirculation can be imaged directly with two-photon microscopy. Clinical TBI, by contrast, is intrinsically heterogeneous—focal and diffuse injuries vary, patient physiology is perturbed by sedation, surgery, systemic shock, and coagulopathy (4)—and direct microvascular imaging is unavailable. These differences mean that mechanisms established in the laboratory, however rigorous, cannot be assumed to operate identically in patients; their relevance must be validated in the clinical context. This is not an argument against the stalling concept but a prescription for how to develop it: the experimental findings define what to look for, and the clinical tools described in Sections 6 and 7 define how it might eventually be detected and tested in patients.

### 9.4 Relationship with microthrombosis and no-reflow

An important conceptual question is whether capillary stalling is distinct from established phenomena such as microthrombosis and no-reflow. These concepts overlap but are not identical. Microthrombosis describes structural occlusive plugs within vessels, whereas stalling describes a dynamic, often structurally independent, and potentially reversible cessation of RBC transit (40) that can occur without a formed thrombus. No-reflow describes failure of perfusion to recover after ischemia despite upstream recanalization; stalling instead describes ongoing flow dynamics (14, 47) and can, in principle, occur without a preceding ischemic-reperfusion episode. These distinctions have practical consequences because the candidate mechanisms include pericyte constriction, leukocyte plugging, rheological failure, and thrombosis (31, 45, 48), and the optimal detection strategy may require dynamic flow imaging rather than static histology alone. Framing stalling in this way is useful only if future studies demonstrate that it adds mechanistic, diagnostic, or therapeutic information beyond existing microvascular concepts.

### 9.5 Is the macro–microcirculatory uncoupling hypothesis clinically testable?

The hypothesis that macrocirculatory normality can coexist with microcirculatory failure is, in principle, directly testable, and its testability is one of the strengths of this framework. What is required is the simultaneous acquisition of ICP, CPP, PbtO₂, CBF, microdialysis, and—ideally—high-resolution perfusion imaging in a cohort of severe TBI patients (87, 105), followed by analysis of whether a pattern of normal CPP and normal CBF together with low PbtO₂ and metabolic crisis occurs, and whether this pattern is associated with markers of microvascular dysfunction and with worse outcome. Demonstration that such macro–micro uncoupling exists, is common, and is prognostically meaningful would convert the hypothesis from a theoretical construct into a validated clinical entity, and would provide the basis for microcirculation-guided therapy. The essential step is to look for the signature of microvascular failure in patients whose macrocirculation appears adequate—precisely the population in whom the conventional paradigm currently offers reassurance.

### 9.6 Therapeutic dilemma: reperfusion versus hemorrhage

The therapeutic program outlined in Section 8 confronts an important safety dilemma. Restoring capillary patency through antithrombotic, NET-directed, or antiplatelet strategies could improve tissue oxygen delivery, but in patients with traumatic intracranial hemorrhage the same interventions may increase the risk of hemorrhage expansion (64, 65, 112). This concern is especially relevant during the early post-injury period, when hemostatic stability may be uncertain. Microcirculation-targeted therapies therefore require careful consideration of treatment timing, individual hemorrhagic risk, and intervention selectivity. A potentially safer path may involve cell-specific or microvascular-selective approaches—such as modulation of pericyte tone, leukocyte adhesion, or glycocalyx integrity—that aim to improve capillary perfusion without substantially disturbing systemic hemostasis (13, 54). Defining which interventions can achieve this balance, and in which clinical contexts, is a central translational challenge.

### 9.7 Long-term evolution of TBI and chronic cerebrovascular dysfunction

The discussion above has centered on acute and subacute secondary injury, but the evolution of TBI is increasingly recognized to extend over years. Human studies show that clinical outcomes after TBI do not simply plateau after the acute phase; rather, a substantial minority of patients experience decline in cognitive, psychiatric, and functional status years after injury, supporting a view of TBI as a chronic disease process (119). Complementing this clinical picture, experimental studies demonstrate that social-behavioral deficits in mice emerge in parallel with delayed reductions in cerebral blood flow and vascular density months after injury, suggesting that chronic cerebrovascular dysfunction—potentially including persistent microcirculatory abnormalities—may contribute to long-term disease progression (120). These observations extend the relevance of capillary stalling and microvascular failure beyond the acute window and underscore the importance of defining the chronic trajectory of microcirculatory dysfunction after TBI.

### 9.8 Future research priorities

Five priorities should guide the field. Priority 1 is to confirm whether capillary stalling actually occurs in human TBI, using whatever direct or high-resolution imaging approaches can be applied to the clinical setting. Priority 2 is to determine the relative contributions of the candidate mechanisms—neutrophils, pericytes, platelets, and red blood cells—so that therapeutic effort is directed at the dominant driver. Priority 3 is to define the temporal evolution of stalling after injury, identifying when it begins, whether it resolves or persists, and whether there is a window for intervention. Priority 4 is to develop clinically applicable markers or imaging methods that can detect microvascular failure at the bedside, converting the concept into a monitorable quantity. Priority 5 is the decisive one: to determine whether restoring capillary patency improves neurological outcome. Without an answer to Priority 5, the entire framework remains intellectually interesting but clinically unproven.

## 10 Conclusion

Microvascular dysfunction after traumatic brain injury is not fully captured by measures of global perfusion. At the capillary scale, interruption or marked slowing of red blood cell transit may coexist with apparently adequate intracranial pressure, cerebral perfusion pressure, and global cerebral blood flow. On the basis of the available experimental and indirect clinical evidence, capillary stalling may represent an under-recognized contributor to persistent tissue hypoxia and metabolic crisis after TBI; however, direct confirmation in human TBI remains a major unmet need.

Several plausible mechanisms converge on capillary flow failure: endothelial injury and glycocalyx shedding promote leukocyte and platelet adhesion; pericyte contraction can narrow capillary lumina; platelet activation and immunothrombosis can convert dynamic flow interruption into structural occlusion; red blood cell dysfunction can impair rheology; and edema-associated extravascular compression can further compromise already vulnerable microvessels. Together, these processes provide a mechanistic framework linking microvascular flow heterogeneity to impaired oxygen extraction, metabolic stress, inflammation, and secondary cellular injury. This framework also explains why satisfactory macrocirculatory targets do not necessarily exclude clinically relevant microvascular dysfunction.

Future work should prioritize direct demonstration and temporal mapping of capillary stalling in human TBI, validation of clinically feasible surrogates, and interventional studies testing whether restoration of functional capillary perfusion improves tissue oxygenation and neurological outcome.

## Glossary

Ang-2: angiopoietin-2
ASL: arterial spin labeling
ATP: adenosine triphosphate
BBB: blood–brain barrier
CBF: cerebral blood flow
CBV: cerebral blood volume
CPP: cerebral perfusion pressure
CT: computed tomography
CTH: capillary transit-time heterogeneity
CTP: CT perfusion
DAMP: damage-associated molecular pattern
DCE: dynamic contrast-enhanced
DSC: dynamic susceptibility contrast
ET-1: endothelin-1
ICAM-1: intercellular adhesion molecule 1
ICP: intracranial pressure
LPR: lactate-to-pyruvate ratio
LSCI: laser speckle contrast imaging
MAP: mean arterial pressure
MRI: magnetic resonance imaging
MTT: mean transit time
NET: neutrophil extracellular trap
NIRS: near-infrared spectroscopy
NO: nitric oxide
OCTA: optical coherence tomography angiography
PbtO₂: brain tissue oxygen tension
RBC: red blood cell
ROCK: Rho-associated protein kinase
ROS: reactive oxygen species
TAMVI: traumatic axonal and/or microvascular injury
TBI: traumatic brain injury
TCD: transcranial Doppler
VCAM-1: vascular cell adhesion molecule 1
VWF: von Willebrand factor
